# The Predictability of Chinese English as a Foreign Language Students’ Willingness to Communicate Through Teachers’ Immediacy and Teacher–Student Rapport

**DOI:** 10.3389/fpsyg.2021.769424

**Published:** 2021-09-30

**Authors:** Manyuan Cai

**Affiliations:** School of Foreign Languages, Xinyang Normal University, Xinyang, China

**Keywords:** willingness to communicate, teachers’ immediacy, teacher–student rapport, EFL, Chinese EFL context

## Abstract

The teacher–learner relationship is not just a simple action and reaction on both sides of the relationship but a complete exchange that takes shape in the context of the factors that affect it. To understand the factors affecting this relationship, the present study investigated the predictability of Chinese English as a Foreign Language (EFL) students’ willingness to communicate (WTC) through teachers’ immediacy and teacher–student rapport. To conduct the study, 858 EFL students from Xinyang Normal University in Henan province of China were invited to participate in the study. To collect the required data, the researcher employed the Willingness to Communicate Questionnaire, Verbal and Nonverbal Immediacy Questionnaire, and Professor–Student Rapport Scale. Pearson product–moment correlation coefficient and structural equation modeling (SEM) were used to analyze the data. Results revealed that there was a strong positive relationship between teacher immediacy and teacher–student rapport and learners’ willingness to communicate. The findings also demonstrated that teachers’ immediacy and teacher–student rapport were positive predictors of learners’ willingness to communicate. The paper argues that teachers need to enhance their interpersonal relations with their students to make them willing to communicate in their classes.

## Introduction

Language learners are social human beings, and making connections among them is one of the obvious needs (Al-Murtadha, 2019; Chen et al., 2019). When learners speak, they do not take words out of their mouths for any purpose. Their purpose is communication (Zhang et al., 2018; Dewaele, 2019). They enter the learning environment with different and sometimes conflicting cultures and subcultures (Lee and Lee, 2020). They do not just try to express themselves, but their goal is to influence their audience (Nkrumah, 2021). In other words, by talking, they want the audience to understand what they mean. Learners’ Willingness to Communicate (WTC), both in the educational environment and outside it, follows this framework; that is, the purpose of learners’ relationship with each other whether through verbal or non-verbal communication is to influence each other. The need for communication, which according to the self-determination theory is one of the basic psychological needs, refers to the desire for strong and stable interpersonal relationships, connection with others, acceptance by them, and a sense of belonging to them and society (Ryan and Deci, 2017).

With the expansion of the positive psychology movement in the last decade (MacIntyre and Gregersen, 2012; MacIntyre and Mercer, 2014; Seligman and Csikszentmihalyi, 2014; MacIntyre et al., 2016, 2019; Pishghadam et al., 2021a; Wang et al., 2021), psychologists focus on the components of positive action, positive experiences, and adaptive human strengths, such as life satisfaction and hope, optimism, happiness, and well-being. Psychology and positive and negative emotions that are increasingly expanding now have attracted the special attention of psychologists (Pavot and Diener, 2008). Positive emotions are associated with extroverted personality traits, and negative emotions are associated with neuroticism (Kalokerinos et al., 2015). Positive emotions are associated with constant physical activity, adequate sleep, social interaction with close friends, and striving for goals; thus, positive emotions may increase during regular physical activity, having a good sleep pattern, having friendly relationships, and having valuable goals.

Teachers and students spend at least a quarter of their daily time in school, and effective communication between them improves their sense of events, processes related to the learning environment, and the quality of teaching and learning (Derakhshan, 2021). Giving importance to interpersonal relationships (teacher–student) and how to improve it might have a significant impact on the quality of second or foreign language teaching (Denies et al., 2015; Dewaele and Pavelescu, 2021). Teachers are one of the most important pillars of society, and in fact, the first adult other than parents who allow this supportive relationship to the students and from this point of view, the quality of communication with them is very important (Dörnyei and Ushioda, 2021).

Teachers in an English as a Foreign Language (EFL) educational setting are one of the most important curriculum facilitators. Their logical (strategic) and psychological (moral) actions form the main elements of teaching. Logical or strategic actions include activities, such as defining, demonstrating, explaining, correcting, interpreting, and evaluating (Sato and Dussuel Lam, 2021). Psychological or moral actions include tasks, such as motivating, persuading, rewarding, punishing, and planning. In addition to logical and psychological actions, teachers also refer to ethical teaching actions in which the teachers demonstrate characteristics, such as honesty, courage, tolerance (Pishghadam et al., 2021b), compassion, respect, and fairness (Lee et al., 2019). Communication with teachers and the quality of their acceptance improve students’ motivation in academic activities (Fallah, 2014), and their emotional and social performance (Henry and Thorsen, 2018). Conflict in this relationship, by creating a sense of insecurity and pressure, is considered an obstacle to its growth. On the other hand, a positive teacher–student relationship motivates the teacher to pursue student progress even by devoting too much time to the job (Hiver and Al-Hoorie, 2020).

The positive consequences of this interaction have been confirmed in several studies (Derakhshan et al., 2019; Pishghadam et al., 2019; Fathi et al., 2021). Positive teacher–student relationship can be effective in an emotional and social relationship of the class (Derakhshan et al., 2020; Xie and Derakhshan, 2021), motivation to learn (Arens et al., 2015), attachment to the learning environment (Chow et al., 2018), academic achievement (Hussain et al., 2021), creativity (Henry and Thorsen, 2018), satisfaction (Hiver and Al-Hoorie, 2020), reduced bullying (Sato and Dussuel Lam, 2021), cooperation in class activities (Fredrickson and Joiner, 2018), learning engagement (Hiver and Al-Hoorie, 2020), hard work in the face of problems (Sato and Dussuel Lam, 2021), friendly help and support (Hiver et al., 2021), understanding of behavior between individualism (Munezane, 2015), creating responsibility (Lee et al., 2019), better behavior in the learning environment (Cao, 2014), freedom in learner work (Denies et al., 2015), and greater classroom attendance (Fredrickson and Joiner, 2018). In general, numerous evidences indicate that the positive teacher–student relationship changes the well-being of teacher and student and is a basis for the next social relationship of the student (Derakhshan et al., 2020).

These positive consequences are explained by relying on exchange theories and evolutionary systems that believe that the source of evolutionary change is the interaction of individuals and the context. Also, the analysis of student transformation is done in the context of different relationships that are exchanged with the student in different dimensions and sizes. As Cao (2014) stated, proximal processes such as the interaction that takes place between the student and the teacher over a period of time are considered to be the primary factor influencing the development of any individual. The teacher–students relationship is also called teacher–student rapport in many studies (Lee, 2020). Closely related to the notion of teacher–students’ relationship or teacher–students’ rapport is teachers’ immediacy that has attracted the attention of EFL and ESL researchers. Researchers have considered immediacy as a set of behaviors that creates an understanding of the physical or mental closeness between teachers and students. They believe that the use of these behaviors increases the psychological distance between them. In general, these behaviors are classified into two categories, verbal and non-verbal. But most researchers have focused on the non-verbal one (Reid and Trofimovich, 2018). Many believe that teacher immediacy might improve EFL/ESL students’ willingness to communicate and, in consequence, might improve their language achievements.

MacIntyre et al. (2011) see teachers’ expectations of learners as an influencing factor in learners’ academic achievement. Munezane (2015) points to the relationship between teacher self-efficacy and learners’ achievement. In addition to teacher expectations and self-efficacy, teacher emotional and educational support for learners leads to teacher–student interaction that might affect their academic achievements and success. In fact, social relations within the classroom are considered as important aspects of the classroom due to their educational, behavioral, and social consequences, so emotions are an integral part of educational activities (Pawlak et al., 2016). Teachers can build a deeper emotional connection with their learners and get to know them and engage them in their classroom activities and solve their problems in the academic and non-academic contexts (Peng, 2020).

According to Reid and Trofimovich (2018), teacher–student rapport and teacher immediacy are a key part of successful teaching and learning. Obviously, EFL learners need their teachers’ support in every learning environment. In other words, teachers are a safe haven for students against problems and a guide to discovering and experiencing the world around them. Yashima et al. (2018) also highlighted the quality of teacher–student rapport and teacher immediacy in the classroom and its particular importance for the learners’ success. In other words, students who have a warm and intimate relationship with their teachers might have high self-confidence (Zhang et al., 2018), interest in their teacher, more motivation to learn (Fredrickson and Joiner, 2018), a positive attitude toward school and enjoy the acceptance of their peers and classmates (Derakhshan et al., 2020). They have pointed out that positive teacher–student rapport and teacher immediacy may protect learners from adverse learning environments, including negative and inappropriate teacher–students relationships. Therefore, teacher–student rapport and teacher immediacy are non-stop and intricate processes in which the perspective and perceptions of the teachers as the main actor and a responsible factor play a key role. Teachers’ personality, beliefs, characteristics, and even their perceptions of students influence what happens in their classrooms and in their interactions in the classrooms. Although the importance of the teachers’ perspective has received little attention in research in this area, this partnership is essential to improve and protect a positive teacher–student rapport and teacher immediacy. In this way, teachers can see themselves through the perspective of students in this mutual relationship and through a reflective look (Pawlak et al., 2016; Wang and Derakhshan, 2021).

Despite the significant function of communication in second and foreign language learning, many learners of EFL who are studying English around the world are often unable to communicate. Chinese EFL learners are no exception. Chinese language learners seem to be very sensitive to the judgment of teachers and classmates about their language abilities. As a result, they are less likely to engage in classroom communication (Wen and Clément, 2003). They also stated that the desire of Chinese teachers to play an authoritative role in educational environments could be another important factor in the inability to communicate between Chinese learners. Inability to communicate in EFL classrooms is considered a negative feature because it hinders the development of communication skills as well as learners’ language achievements, especially in educational environments where the classroom is the only place to communicate in a foreign language. With all these descriptions, it can be argued that teacher–student rapport and teacher immediacy play a key role in learners’ motivation, learning, and success. While many studies focused on the role of interactions between teachers and students in the classroom on academic achievement, there are still some points that remain unclear and questionable and focusing on this notion seems necessary. In addition, interaction and communication in the classroom and any educational setting are often a reflection of the cultural, historical, and social context. Accordingly, there are various approaches to the study of teacher–student interaction that are directly related to the context in which teaching takes place. Therefore, the present study investigated the predictability of Chinese EFL students’ willingness to communicate through teacher–student rapport and teachers’ immediacy.

## Review of Literature

### Teachers’ Immediacy and Teacher–Student Rapport

The notion of teachers’ immediacy that was developed by Mehrabian (1969) has received special attention in recent years. Classified teachers’ immediacy as verbal and non-verbal activities, many researchers explored its importance in EFL and ESL contexts and found that it can affect different aspects of language teaching processes (Derakhshan, 2021). Verbal immediacy refers to any activity that teachers perform to enhance learners’ engagement and motivation. Providing immediate feedback, conversation before and after class, and engagement in learners’ conversations are among the examples of verbal immediacy. Non-verbal immediacy refers to any activity that teachers might perform to reduce their physical or psychological distances with their students. Body language, teacher gestures, and smiling are some of the non-verbal immediacy that might influence teacher–students relationships (Peng, 2020).

Teacher–student rapport is teachers’ responsibility to create an interesting and motivating learning environment. Creating a positive learning environment that learners feel comfortable has a significant impact on learners’ engagement and their wiliness to communicate (Reid and Trofimovich, 2018). Many studies investigated the relationship among teacher immediacy, teacher–student rapport, and learners’ motivation, learners’ engagement, satisfaction, and their learning outcomes (Hsu, 2010). In recent years, some studies have focused on recognizing the teacher–student relationship as a contextual topic (Lee et al., 2019). Burke-Smalley (2018) argued that culture and context might impact teacher immediacy and teacher–student rapport and their willingness to communicate in EFL contexts.

### Willingness to Communicate

Willingness to communicate is to have the choice to speak or to remain silent in a conversation as well as to be ready to enter the conversation at the desired time and with the intended person (Eysenck et al., 2007). Recently, due to the importance of willingness to communicate in improving language learners’ communication ability, it has received special attention of many EFL researchers (Zhang et al., 2018). MacIntyre (2007) defines WTC as the probability of starting a communication, with the ability to choose and the opportunity to start or end it. In his view, conflicting processes are the driving forces and inhibitors of starting a communication. These processes can motivate learners and lead them to effective learning or stop learning by engaging them in emotional factors such as anxiety. MacIntyre and Legato (2011) argue that at least six important variables can predict learners’ WTC. The variables called “antecedents” are communication anxiety, communication competence, motivation, personality, context and culture, and gender and age. In another study, MacIntyre and Doucette (2010) investigated WTC in the French EFL context and found that learners’ anxiety has a direct and significant impact on learners’ WTC and subsequent engagement in classroom activities.

Joe et al. (2017) highlighted the importance of contextual factors and individual differences and argued that when teachers can satisfy learners’ basicpsychological needs, such as competence, autonomy, and relatedness and when the learning environment atmosphere was encouraging, learners’ WTC will increase significantly. They found that teachers’ support and their respectful manner in the classroom are the key factors that influence learners’ WTC. Khajavy et al. (2018) investigated the role of EFL learners’ emotions of WTC and found that a positive classroom environment decreased learners’ anxiety and enhanced WTC. In a similar study, Dewaele and Dewaele (2018) found that the main predictor of WTC among Spanish, German, and French FL learners is classroom anxiety. In a meta-analysis study, Elahi Shirvan et al. (2019) found that EFL learners’ communicative competence and motivations play a pivotal role in predicting Learners’ WTC. Dewaele and Pavelescu (2021) highlighted the link between Foreign Language Enjoyment, Foreign Language Anxiety, and WTC in Romania. They found that emotions are the main predictors of FL learners’ WTC. They argued that factors, such as anxiety, positive classroom climate, learners’ personality, and contextual factors are factors that influence learners’ emotions.

### Empirical Studies in China

Wen and Clément (2003) investigated the impact of Chinese culture on FL learners’ WTC and found that face concern and teachers’ teaching methods are main factors that influence FL learners’ perceptions and behavior in learning environments. Liu (2005) explored Chinese undergraduate students’ silence in EFL classrooms. By conducting questionnaires, reflective journals, and classroom observation, they found that the main predictors of Chinese FL learners are self-confidence, language learning anxiety, fear of losing face, teachers’ and learners’ personality, and their cultural beliefs. In a parallel study, Peng (2007) focused on cultural variables that influence WTC and suggested that factors such as language learners’ anxiety, learners’ risk-taking ability, communication competence, positive classroom atmosphere, teacher support, and teachers’ and learners’ perceptions might affect FL learners’ WTC. Liu and Jackson (2008) also conducted research on Chinese university learners’ WTC and found that FL learners’ anxiety and low proficiency level are among the most important predictors of learners’ WTC.

In a large-scale exploration, Peng and Woodrow (2010) investigated 579 Chinese university learners’ WTC. They found that FL competence and anxiety can predict their motivation and self-confidence. Generally, they suggested that these four factors directly or indirectly influence Chinese FL learners’ WTC. Considering ecological factors, Cao (2014) studied EFL Chinese and Korean language learners. In this study, he examined learners’ class interactions. Findings of this study showed that emotions, self-confidence, personality, perceived opportunity to communicate, educational environment conditions, such as subject, students’ homework, conversations, teacher, and the number of students are closely related to their ability to communicate. The impact of these factors on the WTC is highly variable. Fu et al. (2012) investigated different factors that might influence Chinese EFL learners’ willingness to communicate. They highlighted the critical role of teachers in enhancing learners’ WTC. They argued that EFL teachers should support learner and offer various learning opportunities for language learners. These opportunities can shape learners’ perceptions, their learning climate, and their WTC capability. Shao et al. (2013) argued that EFL learners’ anxiety is a major hindrance in improving their WTC ability. Recently, Liu (2017) investigated the impact of affective, cultural, and linguistic factors on ESL adult learners’ WTC in China. She found that learners’ anxiety level and length of stay in China in correlation with other factors such as intercultural communication competence and learners’ proficiency level predict Chinese ESL learners’ WTC.

Exploring the variables influencing WTC in English, the review of the literature reveals that numerous variables, such as teachers’ and learners’ socioeconomic status (Liu, 2017), academic self-concept, attitude (Dewaele and Pavelescu, 2021), learning environment’s atmosphere (Heckel and Ringeisen, 2019), and teaching methods (Vandergrift and Tafaghodtari, 2010) can affect learners’ willingness to communicate. However, studies in the Chinese EFL contexts and EFL Chinese learners’ WTC are still at an emerging stage. Besides, even though different variables have been investigated, teacher–student relationship, that might influence learners’ WTC ability in EFL contexts, was not investigated in these studies. Therefore, the present study investigated the predictability of Chinese EFL students’ willingness to communicate through teachers’ immediacy and teacher–student rapport.

#### Research Questions

1. Are there any significant relationships between Chinese EFL teachers’ immediacy, teacher–student rapport, and their EFL students’ willingness to communicate?
2. Do Chinese EFL teachers’ immediacy and teacher–student rapport significantly predict their EFL students’ willingness to communicate?

## Materials and Methods

### Participants

Using convenience sampling, 858 EFL students from Xinyang Normal University in Henan province of China were invited to participate in the study. In the sample, there were 40 male (4.7%) and 818 female (95.3%) students. Most of them are university students, majoring in English language, translation, and commercial English with ages ranging from 17 to 22. To distribute the questionnaires, we used Wenjuanxing, an online questionnaire platform popular in mainland China.

### Instruments

The following instruments were employed in the current study to collect the required data:

#### Willingness to Communicate Questionnaire

The WTC Questionnaire is a 10-item scale that was adopted from McCroskey and Richmond (1987). The questionnaire was developed to measure the participants’ disposition toward starting or ending communication. The items are Likert scale that starts from 1 (Definitely not willing) to 7 (Definitely willing). The reported Cronbach alpha coefficient index is 0.95 (*r*=0.95).

#### Verbal and Nonverbal Immediacy Questionnaire

The questionnaire was adopted from the already validated Verbal and Nonverbal Immediacy Questionnaire developed by Gorham (1988). This questionnaire has 22 items that measure perceptions of teachers’ verbal and non-verbal immediate behaviors on a five-point Likert scale (1=never, 2=rarely, 3=occasionally, 4=often, and 5=very often). Seventeen items measure verbal immediacy and five items measure non-verbal immediacy. To check the reliability of the questionnaire, the researchers conducted Cronbach alpha coefficient. The indexes of this test are presented in the result section (*r*=0.93).

#### Professor–Student Rapport Scale

The questionnaire was the already validated Professor–Student Rapport Scale developed by Wilson et al. (2010). It has 34 items that measure students’ attitudes toward teachers and courses as well as perceptions of learning, and students’ motivation. To check its reliability, the researchers used Cronbach alpha coefficient. The results showed a reliability index of 0.95 (*r*=0.95).

### Procedure

To investigate the predictability of Chinese EFL students’ willingness to communicate through teachers’ immediacy and teacher–student rapport, the researcher distributed the Willingness to Communicate Questionnaire, Verbal and Nonverbal Immediacy Questionnaire, and Professor–Student Rapport Scale to 1,000 EFL students from Xinyang Normal University in Henan province of China. To gather more valid questionnaires, I distributed my questionnaires in Chinese to help those participants have a better understanding of the question items. Out of 1,000 distributed questionnaires, only 858 were returned; therefore, the number of participants was 858 (*N*=858). The collected data were analyzed using the following statistics. The descriptive statistics and Pearson correlations were used to answer the first research question and to uncover the relationships among the variables. Then, employing structural equation modeling (SEM), the researcher explored the predictability of Chinese EFL students’ willingness to communicate through teachers’ immediacy and teacher–student rapport. Several fit indices were used to inspect the goodness of fit of the hypothesized model to the data. The indices were goodness-of-fit index (GFI), adjusted goodness-of-fit index (AGFI), root mean square of approximation (RMSEA), comparative fit index (CFI), incremental fit index (IFI), Tucker–Lewis index (TLI), and normal fit index (NFI).

## Results

Kolmogorov–Smirnov was used test to check the normality of data distribution. The results of the normality test are presented in Table 1.

**Table 1:** The results of K–S test.

	Kolmogorov–Smirnov^a^		
	Statistic	Df	Sig.
Immediacy	0.05	858	0.14
Rapport	0.08	858	0.06
Willingness to communicate	0.07	858	0.09

a Lilliefors significance correction.

The presented value and the results of the Kolmogorov–Smirnov test confirm the normal distribution of the data, so parametric statistics were utilized. The results of descriptive statistics of teacher–student rapport, teachers’ immediacy, and EFL students’ WTC are displayed in Table 2.

**Table 2:** Descriptive statistics of the variables of the study.

	*N*	Minimum	Maximum	Mean	*SD*
Immediacy	858	0	88	55.58	14.19
Rapport	858	66	170	134.47	20.41
WTC	858	10	70	48.43	11.78

The results of Table 2 show that 858 students took part in the current study. The results also indicate that the mean and SD of rapport (*M*=134.47, *SD*=20.41) was more than the mean values for immediacy (*M*=55.58, *SD*=14.19) and WTC (*M*=48.43, *SD*=11.78). The results of Cronbach alpha analyses are summarized in Table 3.

**Table 3:** Results of Cronbach Alpha indexes.

Scale	Sub-scales	Cronbach alpha
	Verbal	0.93
Immediacy	Non-verbal	0.70
	Overall scale	0.93
Rapport		0.96
WTC		0.95

The results of Table 3 represent that the employed questionnaires and their sub-scales have reasonable indexes of Cronbach alpha (more than 0.7).

### Data Analysis for the First Research Question

The first research question raised in the study was:

1. Are there any significant relationships between Chinese EFL teachers’ immediacy, teacher–student rapport, and their EFL students’ willingness to communicate?

To answer the first research question, the researchers employed Pearson correlation. The results of this analysis are presented in Table 4.

**Table 4:** Results of Pearson correlation between overall teachers’ immediacy, teacher–student rapport, and English as a Foreign Language (EFL) students’ willingness to communicate.

		Immediacy	Rapport	WTC
Immediacy	Pearson correlation	1		
	Sig. (two-tailed)			
	N	858		
Rapport	Pearson correlation	0.52^**^	1	
	Sig. (two-tailed)	0.000		
	N	858	858	
WTC	Pearson correlation	0.51^**^	0.40^**^	1
	Sig. (two-tailed)	0.000	0.000	
	N	858	858	858

** Correlation is significant at the 0.01 level (two-tailed).

The relationships between Chinese EFL teachers’ immediacy, teacher–student rapport, and EFL students’ willingness to communicate were investigated using Pearson product–moment correlation coefficient. To conduct the test, preliminary analyses were carried out to ensure no violation of the assumptions of normality and homoscedasticity. The results of Table 4 indicate that the null hypothesis was rejected and there is a positive significant relationship between overall immediacy and rapport (*r*=0.52, *n*=858, *p*=0.000, and *α*=0.01), immediacy and WTC (*r*=0.51, *n*=858, *p*=0.000, and *α*=0.01), and WTC and rapport (*r*=0.40, *n*=858, *p*=0.000, and *α*=0.01).

The results of Pearson correlation between two sub-constructs of immediacy and overall teacher–student rapport and EFL students’ WTC are presented in Table 5.

**Table 5:** Results of Pearson correlation between two sub-constructs of immediacy and overall teacher–student rapport and EFL students’ willingness to communicate.

	Verbal	Non-verbal
Rapport	0.51^**^	0.50^**^
WTC	0.45^**^	0.44^**^

** Correlation is significant at the 0.01 level (two-tailed).

Pearson product–moment correlation coefficient was employed to investigate the relationship between two sub-constructs of immediacy and overall teacher–student rapport and EFL students’ WTC. The results of Table 5 reveal that there is a positive significant relationship between both sub-constructs of immediacy and overall rapport and WTC. The results also demonstrate that rapport has the highest correlation with verbal immediacy (*r*=0.51, *n*=858, *p*=0.000, and *α*=0.01) and WTC has the highest correlation with verbal immediacy (*r*=0.45, *n*=858, *p*=0.000, and *α*=0.01).

### Data Analysis for the Second Research Question

The second research question raised in the study was:

2. Do Chinese EFL teachers’ immediacy and teacher–student rapport significantly predict their EFL students’ willingness to communicate?

**Figure 1:**
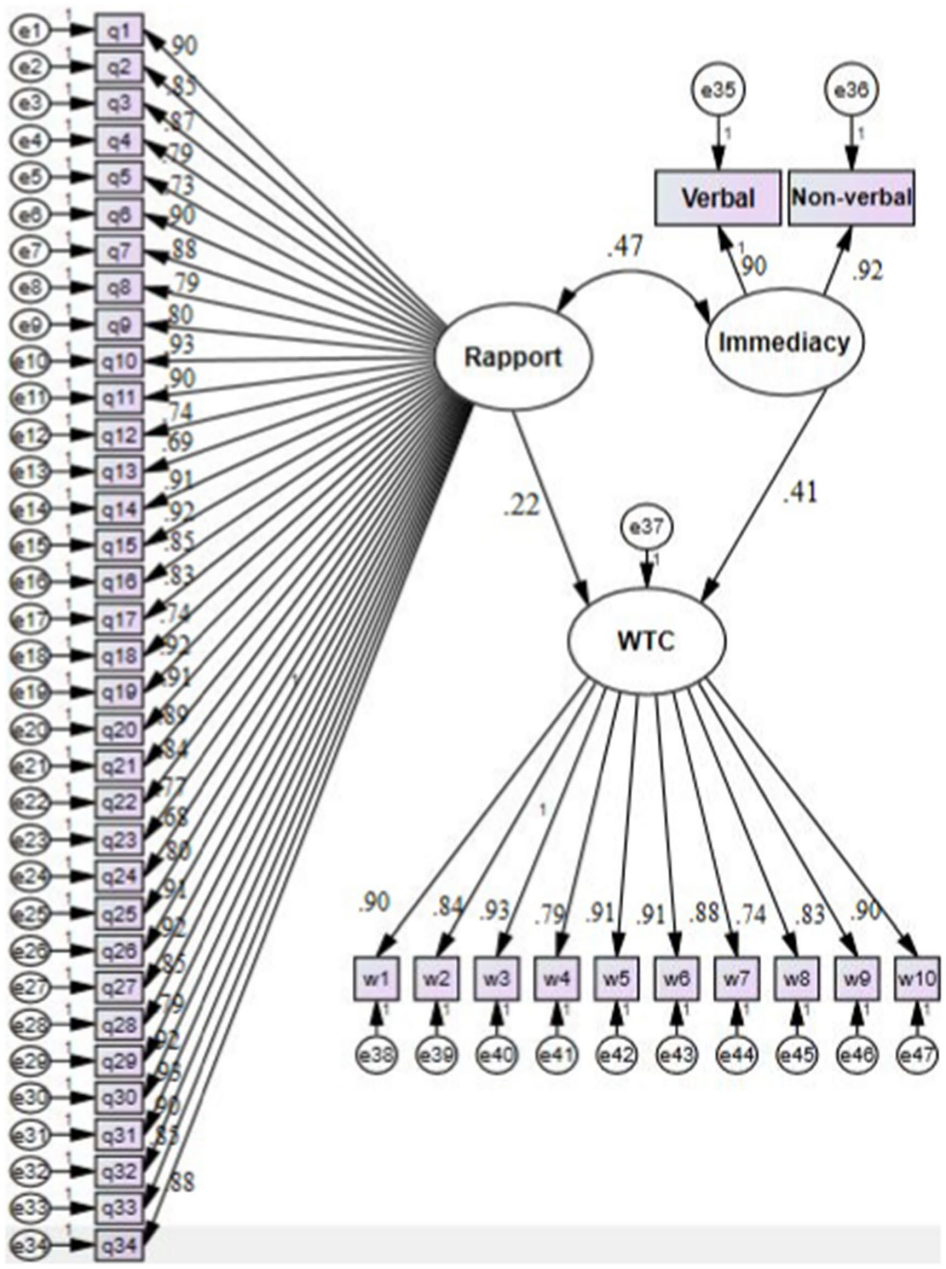
The model of the interrelationships among the variables.

To answer the second research question, the researchers used SEM through Amos (version 24). To check the strengths of the causal relationships among the components, the standardized estimates were observed. The model of the interrelationships among the variables is presented in Figure 1.

The results in Figure 1 demonstrate that both teacher–student rapport (*β*=0.22, *p*<0.05) and teachers’ immediacy (*β*=0.41, p<0.05) are positive predictors of students’ WTC significantly. In addition, the results reveal that teachers’ immediacy correlated positively and significantly with teacher–student rapport (*β*=0.47, *p*<0.05).

To check the model fit, goodness-of-fit indices were employed. Goodness-of-fit indices are presented in Table 6. In this study, χ2/df, GFI, CFI, and RMSEA were employed. To have a fit model, χ2/df should be less than 3, GFI CFI, and NFI should be above 0.90, and RMSEA should be less than 0.08.

**Table 6:** Goodness-of-Fit Indices.

	χ^2^/df	GFI	CFI	NFI	RMSEA
Acceptable fit	<3	>0.90	>0.90	>0.90	<0.08
Model	2.03	0.91	0.93	0.93	0.06

The results of Table 6 reveal that all the goodness-of-fit indices are within the reasonable range. Therefore, the model enjoyed acceptable validity.

## Discussion

The present study investigated the predictability of Chinese EFL students’ willingness to communicate through teachers’ immediacy and teacher–student rapport. The research findings first showed that the dimensions of teachers’ immediacy and teacher–student rapport were directly positive predictors of the willingness to communicate. Second, both dimensions of teacher–students interaction are directly related to each other. The findings related to the first research question of the study indicated that there is a statistically significant and positive relationship among teachers’ immediacy, teacher–students rapport, and their willingness to communicate. Although these findings were empirically and theoretically predictable, we must pay attention to this critical point that according to Table 4 of the correlation matrix, the dimensions of the teacher–student interaction had a very strong correlation with the WTC. The findings also show that there is a stronger correlation between teachers’ immediacy and WTC compared to the teacher–student rapport. It is therefore not surprising that these dimensions have predicted learners’ WTC. Based on the correlation matrix, the variables studied in the present study have shown a logical relationship with each other and have shown their predictive power by being measured in the model. If we take a statistical look at these findings, we can conclude that the dimensions of teacher–student interaction have been influenced by strong predictors of teachers’ immediacy and teacher–student rapport.

Although these findings are consistent with some studies, in relation to the significance of the dimension of teacher support and guidance and academic vitality, this finding can be justified in terms of intercultural differences. Numerous factors, such as economic and social status, individual differences, and the type of classes play a role in the interpersonal teacher–student relationship. Therefore, in classes with a large number of students, the opportunity for direct and supportive communication with the teacher is limited, which in turn can have adverse consequences. Students in a learning environment that provides students with a choice and is supported by a supportive principal or teacher show positive feelings, higher positive emotions, well-being, and constructive relationships. Conversely, it can be argued that whenever teacher–student interactions are fraught with uncertainty and confusion, such relationships are not expected to lead to academic achievement and students’ ability to deal constructively with classroom challenges.

These findings are consistent with the results of MacIntyre and Doucette (2010), Cao (2014), and Dewaele and Pavelescu (2021). In interpreting this finding, it can also be pointed out that the uncertain relationship between teacher and students may be associated with decreased social skills and increased behavioral problems such as aggression and less acceptance of students among peers (Zhang et al., 2018; Lee et al., 2019). Therefore, such interaction with uncertainty and dissatisfaction cannot lead students’ academic achievement and their adaptation to the problems. According to the correlation matrix, the dimension of teacher immediacy has a stronger relationship with WTC. This finding is consistent with the findings of other researchers (Khajavy et al., 2018; Reid and Trofimovich, 2018). Like Dörnyei and Ushioda (2021), the participants answer to the questionnaire study that when the environment is more controlling, more negative emotions are created in students and the controlling conditions create apathy.

In confirming, Liu (2017) highlighted the vital role of the teacher in increasing students’ WTC. He considered that teacher abuse in the classroom is considered a factor in the decline of the student–teacher relationship. Explaining this finding, it can be said that the assertive and controlling behavior of teachers to control the classroom gives them less opportunity to communicate with students. The results of this study also showed that teachers’ social skills are among the important factors that can have a significant impact on students’ WTC (Peng, 2020). The positive relationship between this feature and the positive aspects of students’ learning is shown in the (Heckel and Ringeisen, 2019).

## Conclusion

The current study investigated the relationship among teacher–students rapport, teachers’ immediacy, and their wiliness to communicate. In addition, the study tried to shed light on the predictability of these two factors on WTC. The finding demonstrated that there was a statistically significant relationship between the variables. The findings also confirm that teacher–students rapport and teachers’ immediacy can be a positive predictive of learners’ WTC.

Although many studies emphasize a close relationship between teacher and students, establishing a stable relationship is an intricate process. Generally, it requires working outside the educational environment. As Yashima et al. (2018) have shown, the quality of the teacher–student relationship is essential to understanding students’ WTC and their academic engagement. In a safe explanation, the teacher–student interaction can provide a supportive environment for students to become interested in class and education and to feel involved in classroom activities (Munezane 2015). This support and intimate relationship with the teachers gave them a positive academic attitude, which in turn leads to school satisfaction. Therefore, the students will feel confident and positive about the class and the learning environment. This feeling leads to further growth, academic motivation, and student learning. In other words, students’ involvement is a big step toward forming their will and skills (Pawlak et al., 2016).

### Implications

Several factors are mentioned for teachers to improve their teacher–students relationships. Students’ personal characteristics have a great impact on their communication with the teacher. Therefore, teachers must identify the personal needs and unique characteristics of students and make decisions based on them. The first thing that teachers need to consider in creating a reliable platform for communication is the calmness of the classroom and the stress-free atmosphere. Teachers can solve many problems of the educational environment by using different ways of establishing teachers’ immediacy and teacher–student rapport friendship between teacher and student. Considering that the present study showed that psychological well-being and positive emotion have significant effects on students’ communication, it is recommended to use new methods of counseling, psychology, and training in educational and family settings to form a more appropriate educational environment for students. Efforts should be made to reduce their conflicts and ultimately facilitate their academic and social development. It is also suggested that the teaching of these skills start from teacher training programs to increase teachers’ level of creativity.

### Limitations

The study has some limitations that should be controlled in future studies. The first limitation of the study was its instruments. All of the data were gathered through three questionnaires. In the future study, some interview protocols can be adopted to triangulate the data, which can enhance the quality of my present study. The second limitation is that the study did not consider many factors such as age, educational level, culture, and context, so it is advised to take these variables aforementioned into consideration. The third concern is about the data collection scope. The current study only collected the data from one of the normal universities in the central part of China, although the sample of the current study is quite large. In the future study, the data can be collected from the cross-sectional education contexts to enhance the generalizability of the study.

### Suggestions for Further Studies

The study can be replicated to explore other variables, such as age, sociocultural features, and educational level within the demographic information. The study can be replicated to investigate the same variables within contexts other than EFL contexts. Teachers have different perceptions of their professional freedom that could be highlighted in future studies. Future studies can investigate how EFL teachers develop a strong positive relationship with their students that influence students’ learning. More studies are required to know about how EFL teachers make a decision about the best educational task to increase teacher–students relationships and improve students’ learning.

## Data Availability Statement

The original contributions presented in the study are included in the article/supplementary material, further inquiries can be directed to the corresponding author.

## Ethics Statement

The studies involving human participants were reviewed and approved by the Xinyang College Research Ethics Committee. The patients/participants provided their written informed consent to participate in this study.

## Author Contributions

MC conceptualized, designed the current study, collected the data, and drafted the first manuscript.

## Conflict of Interest

The author declares that the research was conducted in the absence of any commercial or financial relationships that could be construed as a potential conflict of interest.

## Publisher’s Note

All claims expressed in this article are solely those of the authors and do not necessarily represent those of their affiliated organizations, or those of the publisher, the editors and the reviewers. Any product that may be evaluated in this article, or claim that may be made by its manufacturer, is not guaranteed or endorsed by the publisher.
